# Assessing structural integrity of the pyramidal tracts with diffusion spectrum imaging to predict postoperative motor function in pediatric epilepsy patients with hemispherectomy

**DOI:** 10.1186/s42494-022-00115-y

**Published:** 2023-02-09

**Authors:** Huaqiang Zhang, Penghu Wei, Chao Lu, Zhenming Wang, Xiaotong Fan, Yongzhi Shan, Guoguang Zhao

**Affiliations:** 1 Department of Neurosurgery, Xuanwu Hospital, Capital Medical University, No. 45, Changchun Street, Xicheng District, Beijing, 100053 China; 2 Department of Radiology, Xuanwu Hospital, Capital Medical University, No. 45, Changchun Street, Xicheng District, Beijing, 100053 China; 3 Beijing Institute for Brain Disorders, Capital Medical University, No. 10, West Tou Tiao, Outer You’an Men, Fengtai District, Beijing, 100069 China; 4 National Medical Center for Neurological Diseases, Beijing, 100053 China; 5 China International Neuroscience Institute, Beijing, 100053 China; 6 Clinical Research Center of Epilepsy, Xuanwu Hospital, Capital Medical University, Beijing, 100053 China; 7 Beijing Municipal Geriatric Medical Research Center, Beijing, 100053 China

**Keywords:** Diffusion spectrum image, Epilepsy, Hemispherectomy, Motor function

## Abstract

**Background:**

Hemispherectomy is an effective treatment option for patients with drug-resistant epilepsy caused by hemispheric lesions. However, patients often have deterioration of their motor functions postoperatively. Diffusion spectrum imaging (DSI) was reliable in presenting the natural shape of the white matter fibers. At the same time, the natural sprawl pyramid tract (PT) might be more intuitive for predicting postoperative motor functions. Therefore, we assessed the motor functions by the natural shape revealed by DSI tractography.

**Methods:**

Ten children with drug-resistant epilepsy who were candidates for hemispherectomy performed DSI PTs tractography and transcranial magnetic stimulation (TMS) for motor mapping. The motor function was evaluated with muscle strength and hand grasping capability. Pyramidal tract (PT) structural integrity and TMS mapping results were compared between patients who remained stable and those with deteriorated motor functions. Receiver operating characteristic (ROC) curves with PTs asymmetric ratio were analyzed to evaluate DSI tractography diagnostic value.

**Results:**

All patients underwent DSI acquisition, while four patients successfully performed TMS. One patient had no response to TMS until the maximal machine output was reached. Four patients failed to perform TMS due to lacking cooperation. One patient was contraindicated to TMS. DSI successfully reconstructed the sharp angle fan-shaped PTs within the hemisphere. The accurate fiber distribution with fiber termination and thickness within the lesioned hemisphere was replicated with DSI tractography. No significance was found in patients’ age, sex, seizure frequency, or medication between patients with stable or deteriorated postoperative motor functions. DSI effectively predicted postoperative motor function as stable with damaged PTs, mild deterioration with atrophied PTs, and intact PTs with contralateral innervation confirmed by intracranial stimulation. The area under the curve (AUC) of DSI tractography was 0.84. According to ROC, the cut-off value of PTs asymmetric ratio was 11.5% with 100% sensitivity and 75% specificity. The sensitivity and specificity of TMS were 2/3 and 1/2, respectively.

**Conclusions:**

The anatomic integrity of PTs with DSI tractography could effectively predict postoperative motor function after hemispherectomy. This enables neurosurgeons to inform patients and relatives about postoperative motor functions with direct morphological evidence of PTs to help them with their surgical decisions.

**Supplementary Information:**

The online version contains supplementary material available at 10.1186/s42494-022-00115-y.

## Background

Hemispherectomy was described independently by Dandy and Lhermitte as a radical treatment for malignant glioma of one hemisphere [1]. Mckenzie first utilized it in treating drug-resistant epilepsy in 1938, which proved to be an effective treatment option for patients with drug-resistant epilepsy caused by hemispheric lesions. After that, functional anatomy was induced to reduce the complications associated with hemispherectomy [2]. Reported seizure-free rates of hemispherectomy range from 60% to 90% [3, 4].

However, this surgical procedure increases functional defects in the brain. Most patients often have deterioration of their motor functions. Although the ambulatory status of most patients is unchanged or could be returned to presurgical baseline postoperatively, many patients suffer a decrease in muscle strength of the distal part of the arm and hand dexterity [5]. This hemiparesis is often so severe that all active hand functions are lost. Clinically, patients and their families are usually concerned about postoperative motor function defects during counseling. Thus, the prediction of contralateral muscle strength and hand function is an essential step in the preoperative work-up for these patients. This highlights the crucial importance of optimal motor mapping before hemispherectomy.

As functional approaches, transcranial magnetic stimulation (TMS), functional magnetic resonance imaging (fMRI), and magnetoencephalography (MEG) were adopted for motor function localization. Nevertheless, the functional approaches have been denounced either for false activation of undesired movements or for the complex tasks difficult for toddlers to accomplish. At the same time, sedation would alter cerebral activity patterns [6]. As structural approaches, diffusion images could enable the identification of the status of the motor pathway without any tasks, and diffusion scalars such as fractional anisotropy (FA) and the fiber number within corticospinal tracts were reported to be higher on the affected side in patients whose postoperative muscle strength was reduced [7]. Nevertheless, direct morphological criteria such as the shape and the sprawl of the fibers for motor evaluation before hemispherectomy were not reported because of the unquantified property of the diffusion tensor imaging (DTI) technique.

Diffusion spectrum imaging (DSI), a high angular resolution diffusion technique, can define more complex structures than DTI. Studies have shown that DSI could solve the cross and termination problem of the tractography [8], which was the disadvantage of DTI fiber reconstruction. In this report, we assessed the anatomic integrity of PTs with DSI to predict postoperative motor function in epilepsy cases candidate for hemispherectomy. We aimed to provide preliminary data on whether DSI is effective in motor mapping in clinical practices.

## Methods and materials

### Subjects

Drug-resistant epilepsy patients candidate for hemispherectomy were retrospectively recruited from the Neurosurgery Department of Xuanwu Hospital (Beijing, China) between March 2019 and November 2020. A multidisciplinary team of neurosurgeons, epileptologists, pediatricians, neurophysiologists, radiologists, and neuropsychologists carried out the presurgical evaluation. Children with hemiplegic epilepsy because of congenital or acquired hemispheric lesions and those who failed to control the disabling with fair trials of tolerable anti-seizure medications (ASMs) underwent hemispherectomy. Since hemispherectomy is the only and highly effective therapy to achieve seizure freedom of Rasmussen encephalitis [9], patients with Rasmussen encephalitis were excluded from this study. Lesion resection was reckoned to be a better functional outcome when preoperative motor mapping showed that only the lesioned hemisphere innervated the paralytic limbs. This study was approved by the Medical Ethics Committee of Xuanwu Hospital.

### DSI image acquisition

DSI studies were performed on a 3.0 T scanner (GE SIGNA™ Premier, Chicago, USA) using a 48-channel head coil for all patients. A novel head stabilizer device was used to prevent head motion. In this trial, DSI data were acquired with equal spacing in q-space on a Cartesian grid in 259 directions, with a maximum *b*-value of 7000 s/mm^2^. The scan parameters were as follows: repetition time (TR) = 5548 ms, echo time (TE) = 84.1 ms, field of view (FOV) = 224 × 224 mm^2^, and voxel size = 2.0 × 2.0 × 2.0 mm^3^ voxels. The flip angle was 90°. When the DSI data were acquired, they were analyzed by an experienced radiologist.

### DSI tractography

For the tractography of PTs, Digital Imaging and Communications in Medicine (DICOM) data were imported to DSI Studio (http://dsi-studio.labsolver.org). Reconstruction was performed by the generalized q-sampling imaging (GQI) method with orientation distribution functions output [10]. The diffusion sampling length with cerebral spinal fluid (CSF) calibration was 1.1. Before fiber reconstruction, regions of interest (ROIs) were drawn in orientation distribution function map images by a neurosurgeon with 20 years of experience within 3 days. The two overlapping parts were the final ROI.

An ROI was drawn at the precentral gyrus, cerebral peduncle, and medulla to guide tractography to perform the fiber tracking for a given lateral section of the PT. The PT was highlighted in the two-dimensional quantitative anisotropy (QA) map at these regions. A threshold was set at a value where the orientation signal best matches the profile of the structural T1 images. The angular threshold was 80 degrees, the step size was 0.4 mm, and the smoothing was 1.0. After reconstructing fiber tracts with DSI data, the PT structural integrity of motor fibers originating from the precentral gyrus was traced to the ventral horn of the spinal cord at the level of the occipital foramen area. Based on the PT and cerebral peduncle asymmetric study with poststroke hemiparetic patients, which showed the average affected/unaffected ratio was 72% [11], the atrophy PTs in the current study were defined by 72% or fewer fiber numbers of the PT compared to the unaffected side. Damaged PTs referred to those with non-continuous fibers between the precentral gyrus and medulla, whereas the symmetric PTs were considered intact.

### Pre and post-surgical evaluation

The preoperative evaluation comprised comprehensive seizure history, neurological examination, neuropsychological examination, long-term video electroencephalography, and neuroimaging. Since TMS was a commonly used technique, which could generate preoperative motor maps [12]. Children who had no contraindications also received a TMS motor mapping. The patients were tested sitting on their parent’s lap, allowing them to watch a video or play with their toys. The TMS was performed with a simple figure-8 coil connected to a magnetic generator. The stimulation was applied to the Rolandic cortex, with an intensity initially set at 50% and an increment of 10% of the machine output. The motor-evoked response (MEP) elicited by TMS was recorded by surface electromyography (EMG) from the bilateral abductor pollicis brevis. The stimulation was delivered until the EMG response was induced or the maximal output was reached.

Considering that complex clinical motor function examination may be difficult for children, the motor function was evaluated by the muscle strength with Medical Research Council (MRC) score and a simple grasping skill according to the Melbourne Assessment before surgery and at hospital discharge. A well-grasping ability was categorized with a fluent, accurate grasp of the pellet. The motor function was classified as “pre/postoperative no grasp,” “grasping preserved,” or “grasping lost.” were evaluated preoperatively and at hospital discharge.

Hemiplegic patients with congenital malformation, dysplasia, or destructive lesions in the unilateral hemisphere that resulted in drug-resistant epilepsy underwent anatomic hemispherectomy. For those whose motor functions were normal, while TMS and bilateral robust intact PTs indicated unilateral innervation of upper limbs, stereoelectroencephalography (SEEG) investigation was considered for a better functional outcome with lesion resection.

### Surgical procedure

Patients underwent pathologic hemisphere removal with the approach of peri-insular hemispherectomy. The patient was supinely positioned, with a question mark skin incision. Under the neurosurgical microscope, the surgeon entered the lateral ventricle with an infra-insular window, followed by resecting the frontal, temporal, and occipital lobes along the circular sulcus. The mesial temporal structures, including the amygdala and hippocampus, were removed. Insular cortex resection was completed by subpial aspiration.

### Statistical analysis

Statistical analyses were performed using GraphPad Prism version 9.0 (San Diego, USA). Student’s *t*-test and the Mann-Whitney U test were applied to identify the difference in clinical data between patients who were stable and those who deteriorated in motor function postoperatively. ROC curves were obtained and analyzed to identify the usefulness of motor mapping with DSI tractography. Sensitivity and specificity were compared between DSI tractography and TMS.

## Results

### Patients

We enrolled ten patients (age range: 2–14 years, mean age: 6.8 years, female: 5, male: 5) with hemispheric lesions who completed clinical assessment and DSI tractography (Table 1). None of the patients had any complications due to the diffusion MRI scan. One patient received SEEG investigation since the preoperative evaluation highly suspected that the patient’s motor function was unilaterally dominated. The SEEG recordings showed that the patient’s seizures originated from the right frontal lobe. The intracranial stimulation with SEEG electrodes in the right precentral gyrus elicited tonic contraction of his left hand. Since the presurgical evaluation suggested a strong innervation of the right precentral gyrus in his left hand, the patient received removal of the right temporal, parietal, occipital, and frontal lobes anterior to precentral sulcus, leaving the precentral gyrus in the suit. All of the other patients underwent hemispherectomy. All patients were seizure-free since the surgery. There was no mortality and no severe postoperative complication requiring further surgical intervention in the study collective. No significance (*p* > 0.05) was found in patients’ age, sex, seizure frequency, or medication between the groups who remained stable or deteriorated in motor functions postoperatively.

**Table 1:** Clinical characteristics of the 10 children undergoing hemispherectomy

pat	age	sex	seizure frequency	ASMs	lesioned hemi	PT structural integrity		TMS	surgery	MRC score		grasping ability	motor function outcome
						healthy hemi	lesioned hemi			pre-OP	post-OP		
1	2	M	daily	2	L	robust	damaged	failed	hemispherectomy	4	4	pre/post no	stable
2	10	M	daily	4	L	robust	damaged	ipsi-lateral	hemispherectomy	4	4	pre/post no	stable
3	5	M	daily	3	L	robust	damaged	contra-lateral	hemispherectomy	4	4	preserved	stable
4	10	M	daily	4	R	robust	damaged	contraindicated	hemispherectomy	4	4	pre/post no	stable
5	7	F	weekly	2	L	robust	damaged	failed	hemispherectomy	4	4	preserved	stable
6	5	F	daily	3	R	robust	atrophied	ipsi-lateral	hemispherectomy	4	3	pre/post no	deteriorated
7	5	F	daily	4	L	robust	atrophied	failed	hemispherectomy	4	3	pre/post no	deteriorated
8	4	F	daily	3	R	robust	atrophied	failed	hemispherectomy	4	3	lost	deteriorated
9	6	F	weekly	2	R	robust	atrophied	no response	hemispherectomy	5-	2	lost	deteriorated
10	14	M	daily	3	L	robust	robust	contra-lateral	lesioned resection	5	5	preserved	stable

*Abbreviations*: *ASMs* anti-seizure medications, *PT* pyramidal tract, *lesioned hemi* lesioned hemisphere, *healthy hemi* healthy hemisphere, *MRC score* Medical Research Council (MRC) Scale for muscle strength

### Tract fiber distribution and integrity

**Fig. 1:**
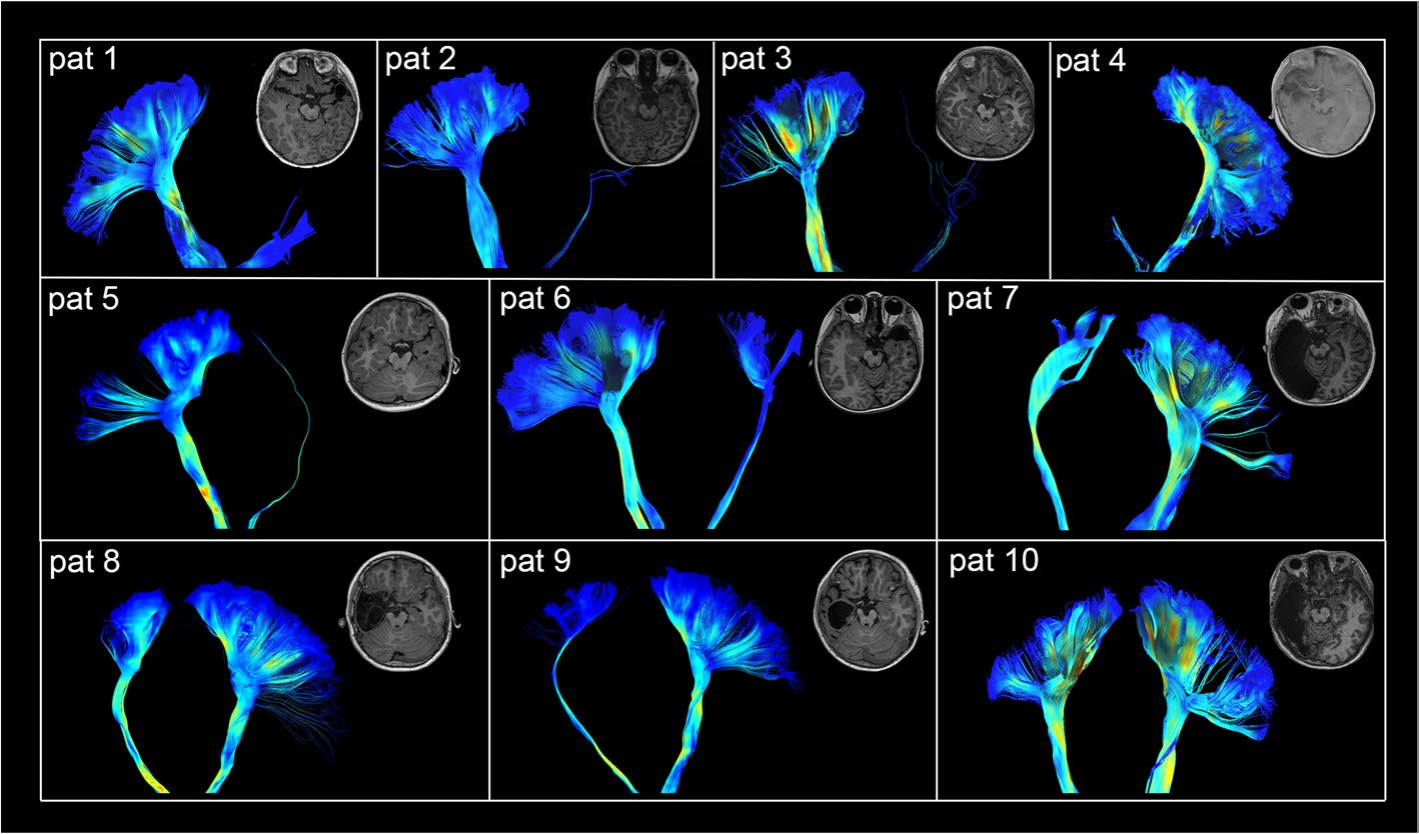
PT tractography of all the patients. Patients 1–5’s PTs were damaged. Patient 6–9’s PTs within the lesioned hemisphere were continuous but severely atrophied between the precentral gyrus and the spinal cord. Patient 10’s PTs were symmetric and robust between the two hemispheres. Abbreviation: pat (patient), PT (pyramidal tract)

For all patients, the PTs within the non-lesioned hemisphere were robustly intact between the precentral gyrus and ventral medulla. DSI tractography reconstructed the sharp angle fan-shaped PT distribution and crossed fibers within the centrum semiovale. Structural origins with a precentral gyrus folding pattern, termination of tract fibers, and anatomic features of motor fiber width and thickness along PTs were accurately replicated. In the lesioned hemisphere, five patients’ PTs were damaged, four patients had continuous but atrophied PTs, and one had almost symmetric PTs between the two hemispheres (Fig. 1).

### TMS mapping

All patients underwent preoperative TMS motor mapping. Five patients successfully underwent TMS, which predicted postoperative motor function in three children. One patient had no response from both hemispheres until the max machine output was reached. The other patient’s TMS result demonstrated that his hand was unilaterally innervated, but he preserved his grasping ability after the hemispherectomy. Four children failed to perform TMS owing to a lack of cooperation. The other patient could not undergo TMS because of a contraindication with a skull defect.

### Postoperative motor function change

**Fig. 2:**
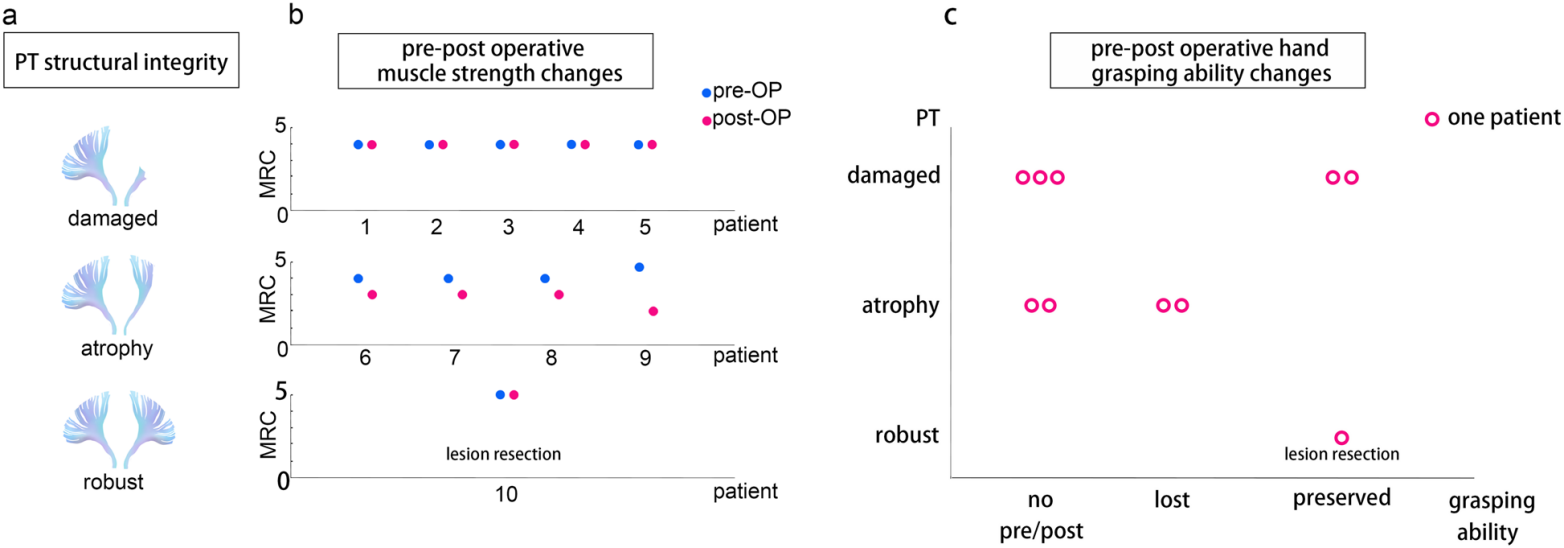
DSI tractography of pyramidal tracts (PTs) and pre to post-operation motor function changes. **a** Illustration of PT tractography result: deficit of all the PT fibers was classified as “damaged”; continuous PTs with a deficit in most of the fibers were classified as “atrophied”; while the PTs within the lesioned hemisphere that were symmetric with the healthy hemisphere PTs were classified as “robust PTs.” **b** Patient 1–5 with broken PTs within lesioned hemisphere exhibited mild paralysis with muscle strength pre-operatively but remained stable postoperatively. Patients 6–9 with atrophied PTs exhibited paralysis with muscle strength pre-operatively and suffered mild deterioration postoperatively. Patient 10 exhibited symmetric and robust PTs between hemispheres, with contra-lateral innervation confirmed by intracranial stimulation. This patient received multilobar lesioned resection and remained stable with muscle strength postoperatively. **c** Three patients with damaged PTs in the lesioned hemisphere had “no grasping ability” pre-operatively. Two of the patients with damaged PTs in the lesioned hemisphere were able to grasp pre-operatively. These patients’ postoperative hand grasping functions were stable compared with preoperative hand function. Four patients with atrophied PTs in the lesioned hemisphere could not grasp after the operation. Two of these patients who could grasp preoperatively lost their grasping ability postoperatively. The patient with robust symmetric PTs received multilobar lesion resection and remained stable in motor function after the operation. Abbreviation: PT (pyramidal tract), pre-OP (pre-operative), post-OP (post-operative)

The MRC motor score and grasping ability were evaluated between the pre-and postoperative state. Among patients who underwent hemispherectomy, five patients with broken PTs within the lesioned hemisphere retained stable motor function postoperatively with an MRC score (Video 1 s a, b, and c in the online-only Supplementary material showed the pre-and post-operative walking of Patients 1, 2, and 3). Of these patients, three patients had no grasping ability pre-and postoperatively. Two hemiparetic patients with grasping capability pre-operatively preserved their hand function after the operation. Four patients with severe atrophy of PTs in the lesioned hemisphere experienced deterioration of muscle strength after hemispherectomy (see Video 1 s d in the online-only Supplementary material for pre-and post-operative walking for Patient 7). Besides, two of these patients whose hand functions were normal lost grasping ability postoperatively. One patient with intact PTs confirmed contralateral innervation of motor function by the intracranial stimulation and underwent lesion removal (Table 1 and Fig. 2). All patients remained seizure-free since hospital discharge.

### Correlation between DSI tractography, TMS, and motor function

**Fig. 3:**
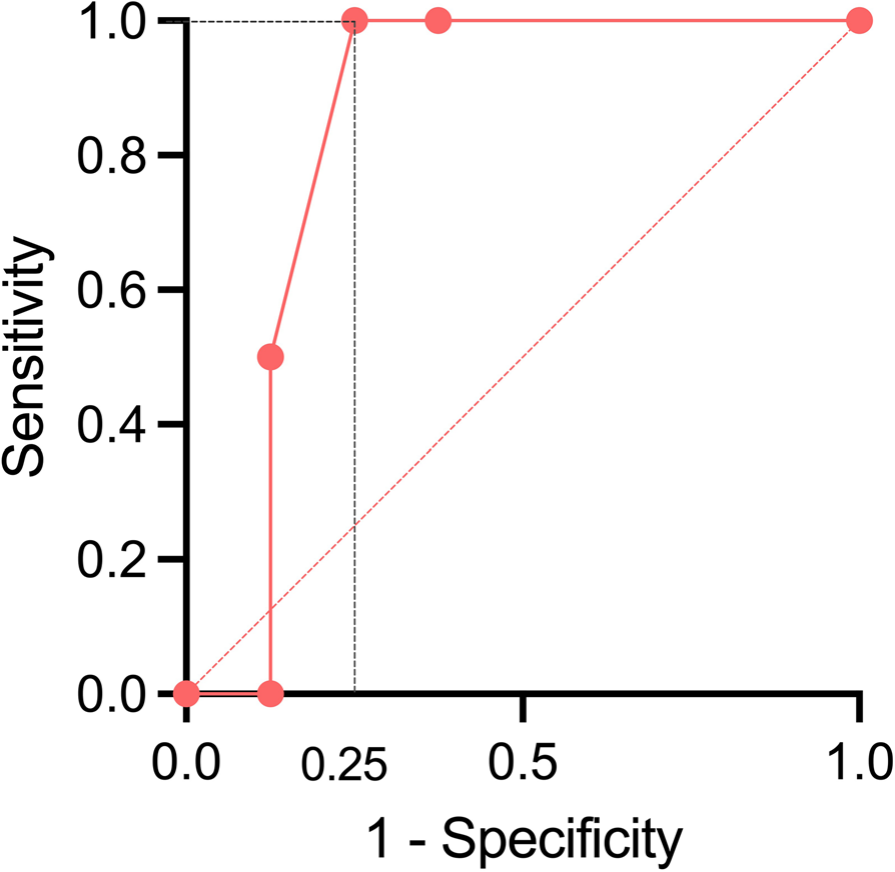
ROC curve of motor mapping with DSI tractography compared with postoperative motor function outcome. Abbreviation: ROC (receiver operating characteristic), DSI (diffusion spectrum imaging)

The ROC of PT structural integrity was obtained to identify the usefulness of motor function mapping with DSI. The area under the ROC was 0.84 (*p* = 0.15), suggesting that DSI tract integrity is a good approach to localizing motor innervation. According to the ROC, the cut-off value of the asymmetric ratio of PTs was 11.5%, leading to postoperative motor deterioration, with sensitivity being 100% and specificity being 75% (Fig. 3). The ROC analysis of TMS was not performed because 6/10 of the subjects failed or contraindicated to TMS or had no response till the maximal output of the machine was reached. Among the subjects with TMS performed, the sensitivity was 2/3, while the specificity was 1/2.

## Discussion

Hemispherectomy has been used successfully for hemispherical drug-resistant epilepsy. A few studies have focused on predicting postoperative motor function with invasive [13] or non-invasive methods without direct morphologic change evidence [2, 14, 15]. Here, we presented a report on the evaluation of residual motor function by direct morphological reconstruction of PTs, which had higher sensitivity and specificity than TMS. With direct morphological evidence being provided, our methodology would be crucial for the caregivers and patients considering surgical decisions for hemispherectomy.

In clinical practice, fMRI, MEG, Wada test, and intraoperative motor mapping can be employed to examine the cerebral function of the intact and lesioned hemisphere. Several authors have documented success using these methods to assess residual motor function. Wang et al. [16] reported 92.9% preoperative fMRI motor mapping sensitivity in healthy children. It was reported that motor cortex mapping in children with epilepsy by resting-state fMRI revealed a sensitivity of 87.5% [17]. MEG is a helpful modality for preoperative localization of the motor cortex. Tarapore et al. [12] reported that MEG imaging motor mapping successfully localized a motor site for index finger flexion comparable with direct cortical stimulation. In comparing the findings with previous studies, the current study suggested good sensitivity with DSI tractography for motor cortex mapping without task cooperation, which may be difficult for children with epilepsy. The feasibility of this approach could be explained by the finding that sensorimotor functional connectivity was PT-dependent in children with unilateral cerebral palsy [18].

DSI mapping predicted the motor function with higher sensitivity and specificity than TMS in the study. Although children under 10 years of age have higher MEP thresholds that decrease to adult levels by mid-adolescence [19], TMS was proven valuable and reliable in eliciting MEPs in children younger than three [20]. However, some patients failed to perform TMS or had no MEP response. Koudijs et al. [21] reported that 14/34 children had no motor responses in a TMS study of motor lateralization in children with epilepsy. The most obvious explanation for the lack of TMS responses in these children may be the antiepileptic medications, which may have had an inhibitory effect that resulted in the elevation of the cortex action threshold. In this study, DSI tractography could more precisely assess the motor function of children with hemispheric epilepsy than TMS mapping. Since DSI tractography does not need the cooperation and interferences of ASMs, it is a possible useful probe in epileptic children’s clinical management.

Several reports have correlated anatomical evidence with human motor function. The PT is the major neuronal pathway that mediates voluntary movements; hence, the preservation or recovery of the PT is mandatory for good recovery of impaired motor function [22]. This may be why most research chooses PTs’ morphologic changes as an indicator for motor function recovery. Studies have reported that patients with a cerebral peduncle asymmetric ratio (contralateral/ipsilateral) > 1.5–2 would obtain an improved or unchanged motor function [3, 23, 24]. Govindan et al. [25] predicted an 8-year-old girl’s motor function with DTI tractography before her hemispheric surgery. Nelles et al. [7] applied DTI fiber tracking in 34 patients, which found that FA of the affected PTs was significantly higher in patients with postoperative muscle-strengths loss than in patients without. Subsequently, Wang et al. [16] reported that bilaterally robust, symmetric PTs on DTI-FA maps were significantly associated with a severe postoperative motor decline in 25 patients. Consistent with these findings, we predicted post-surgical motor function accurately with DSI tractography in the present study. Moreover, DSI could solve the crossing and termination problem, for which fibers from the lateral and medial precentral regions could not be visualized in DTI fiber tracking [26]. DSI tractography could replicate the fan-shaped distribution of PTs between the lateral and superior precentral gyrus, with an accurate representation of the origin and termination of tract fibers and anatomic features of motor fiber width and thickness. Therefore, it could provide more neural pathway information, which may help to better inform patients and relatives about the motor outcomes after surgery.

Patient 10 with a hemispheric lesion involving the frontal, temporal, parietal, and occipital lobes but remains normal in motor function preoperatively. A little part of his precentral gyrus was affected in the infant intracranial hemorrhage due to vitamin K deficiency. However, his PTs remained intact during the development, confirmed by direct electrical stimulation following the SEEG procedure. Previous studies have also suggested that the infarct region but not the total infarction volume was directly linked to the PTs atrophy [11]. This case highlights the importance of preoperative motor function mapping, especially for those with normal motor function before the hemispherectomy. Lesion resection may also be practical after SEEG investigation or an alternative approach for those with predictors of function deterioration after surgery.

There are some limitations in the current study. Our clinical data were based on retrospective analysis of hospital charts, which resulted in a broad classification of motor function. Further, assessing a functional outcome using an anatomical study demands solid justification. Additionally, DTI tractography provides another approach to studying the PT before hemispherectomy with a shorter image acquisition time. The small number of patients and lacking comparative studies with DTI may overstate DSI imaging modality for predicting motor preservation. Therefore, our findings need further validation by more rigorous case-control research with a larger patient group. DSI tractography requires a relatively long time for data acquisition, for which the sedation may limit its clinical application in children. However, DSI tractography seems more suitable and cost-effective than the Wada test and awake craniotomy, which is invasive and difficult to administer in children.

## Conclusions

In summary, the anatomic integrity of PTs with DSI tractography could predict postoperative motor function in children with hemispherectomy. This enables neurosurgeons to inform the patients and their caregivers about the postoperative motor function, which is vital in their surgical decision, with direct morphological evidence. Although it takes a relatively long time to acquire high-quality images, DSI tractography could be helpful for young patients who cannot cooperate with other tests or with patients receiving multiple and high doses of ASMs.

## Supplementary Information

**Additional file 1: Video 1s.** Pre-and post-operative walking status. (a, b, and c) Video records of patients 1,2, and 3 showed equally walking pre-and post-operatively. (d) Patient 7 could carefully walk unassisted. However, the neurological examination revealed that muscle strength decreased by 1 grade on the MRC scale.

## Data Availability

The datasets of the current study are available from the corresponding author upon reasonable request.

## Abbreviations

ASMs: Anti-seizure medications
AUC: Area under the curve
CSF: Cerebral spinal fluid
DICOM: Digital Imaging and Communications in Medicine
DSI: Diffusion spectrum imaging
DTI: Diffusion tensor imaging
EMG: Electromyography
FA: Fractional anisotropy
fMRI: Functional magnetic resonance imaging
FOV: Field of view
GQI: Generalized q-sampling imaging
MEG: Magnetoencephalography
MEP: Motor-evoked response
PT: Pyramidal tract
QA: Quantitative anisotropy
ROC: Receiver operating characteristic
ROIs: Regions of interest
SEEG: Stereoelectroencephalography
TE: Echo time
TMS: Transcranial magnetic stimulation
TR: Repetition time
